# Phytochemical Variability of Essential Oils of Two Balkan Endemic Species: *Satureja pilosa* Velen. and *S. kitaibelii* Wierzb. ex Heuff. (Lamiaceae)

**DOI:** 10.3390/molecules27103153

**Published:** 2022-05-14

**Authors:** Valtcho D. Zheljazkov, Ivanka B. Semerdjieva, Charles L. Cantrell, Tess Astatkie, Milica Aćimović

**Affiliations:** 1 Department of Crop and Soil Science, Oregon State University, Corvallis, OR 97331, USA; 2 Department of Botany and Agrometeorology, Faculty of Agronomy, Agricultural University, 4000 Plovdiv, Bulgaria; v_semerdjieva@abv.bg; 3 Department of Plant and Fungal Diversity and Resources, Institute of Biodiversity and Ecosystem Research, Bulgarian Academy of Sciences, 1113 Sofia, Bulgaria; 4 Natural Products Utilization Research Unit, Agricultural Research Service, United States Department of Agriculture, University, MS 38677, USA; charles.cantrell@usda.gov; 5 Faculty of Agriculture, Dalhousie University, P.O. Box 550, Truro, NS B2N 5E3, Canada; astatkie@dal.ca; 6 Institute of Field and Vegetable Crops Novi Sad, Maksima Gorkog 30, 21000 Novi Sad, Serbia; acimovicbabicmilica@gmail.com

**Keywords:** Balkan endemic, Satureja, Bulgaria, chemotypes, intrapopulation, interpopulation

## Abstract

*Satureja pilosa* and *S. kitaibelii* (Lamiaceae) are Balkan endemic plant species, and the composition of their essential oil (EO) is highly variable. The aim of the present study was to establish: (1) the EO variability in two populations of *S. pilosa* (the intrapopulation), and (2) the EO variation in *S. kitaibelii* between nine populations (interpopulation) from Bulgaria and two from Serbia. The EOs of two *Satureja* species were obtained from aboveground plant parts by hydrodistillation and were analyzed by GC/MS/FID. Overall, the EO yield on the intrapopulation level of *S. pilosa* varied from 0.54% to 2.15%, while the EO of *S. kitaibelii* varied from 0.04% to 0.43% (interpopulation). The EO of *S. pilosa* was found to contain thymol and carvacrol as the main constituents, with other major constituents being *p*-cymene and *γ*-terpinene. *S. pilosa* samples in both studied populations formed six chemical groups. The major constituents (*p*-cymene, terpinen-4-ol, bornyl acetate, *γ*-muurolene, *endo*-borneol, *cis*-*β*-ocimene, *trans*-*β*-ocimene, carvacrol, *α*-pinene, thymoquinone, geranial, geranyl acetate, spathulenol, and caryophyllene oxide) of *S. kitaibelii* EO were considered for grouping the populations into ten chemotypes. The present study is the first report on the interpopulation diversity of *S. kitaibelii* EOs in Bulgaria. It demonstrated variability of the EOs between and within the populations of *S. kitaibelii* from Bulgaria. This study identified promising genetic material that could be further propagated and developed into cultivars for commercial production of *S. kitaibelii* and *S. pilosa*, thereby reducing the impact of collection on wild populations.

## 1. Introduction

Bulgarian flora is characterized by a diversity of plant species, including Bulgarian and Balkan endemics [1]. This is due to the specific geographical location of Bulgaria and its geological history [2]. *Satureja pilosa* and *S. kitaibelii* (Lamiaceae) are Balkan endemic species and both have a limited distribution [3]. *Satureja kitaibelii* (*S. montana* ssp. *kitaibelii*) is spread on stony poor soils in the northern part of Bulgaria (Znepole region; Sofia region; the Northern part of the Balkan Mountains), while *S. pilosa* is spread on rocky habitats in the Central and Eastern Balkan Mountains and the Eastern Rhodope Mountains [4]. *Satureja* species are well known as culinary and medicinal herbs in Bulgaria [5]. *Satureja* products are used as natural preservatives for food and in many other industries (perfume, cosmetic, and pharmaceutical industries) [6]. The antimicrobial, antioxidant, anti-hyperglycemic, anti-inflammatory, antifungal, antimicrobial, and anti-mosquito properties of EO from *Satureja* species have been well recognized for many years [7,8,9,10,11,12]. 

*Satureja pilosa* is a species spread in Bulgaria, Greece, and Western Turkey—in regions very close to Bulgaria [12,13,14]. Due to such a narrow area of distribution, there are only a few studies on the phytochemical composition of *S. pilosa* EO. Our previous data showed wide variability in the EO content and composition of *S. pilosa* between different locations and also within a single location in Bulgaria [12]. Furthermore, previous studies identified five chemotypes of the species collected at 33 locations across Bulgaria, namely: (1) thymol and *p*-cymene; (2) thymol, *p*-cymene and *γ*-terpinene; (3) carvacrol and *p*-cymene; (4) carvacrol, *p*-cymene and *γ*-terpinene; and (5) carvacrol [12]. In Bulgaria, *S. pilosa* is of industrial interest as a source of EO, but there is large variation in EO composition [12]. So far there have been no investigations on EO variability of *S. pilosa* at an intrapopulation level. Studies of the diversity of the EO composition of *S. pilosa* are important in order to identify clones that can be propagated and investigated under cultivation. 

The above-mentioned *S. kitaibelii* is an endemic species of the Central Balkan Mountains (Stara Planina) and is spread in North Bulgaria, Southwest Romania, and Eastern Serbia [3,4,15]. According to the official books of Flora of European and Bulgarian flora, *S. kitaibelii* belongs to the *S. montana* group, and it is classified as a subspecies of *S. montana* (*S. montana* subsp. *kitaibelii*) [3,4]. However, according to the International Plant names Index [16], its accepted name is *S. kitaibelii* (https://www.ipni.org/n/884192-1, accessed on 15 June 2021), a separate species. One of the first studies on *S. kitaibelii* of the Bulgarian flora was conducted by Konakchiev and Tsankova [17], who found that the major compounds of the EOs in the studied Bulgarian population were limonene (15.7%) and *p*-cymene (13.1%). However, the authors in the latter study investigated one population using a single sample per population; therefore, the results may not be representative. Thus far there have been no studies on the variability on EO compositions of *S. kitaibelii* in Bulgarian populations. 

The main phytochemical studies on the variability of EO compositions of *S. kitaibelii* were conducted at populations in Serbia and the former Yugoslavia [7,15,18,19] (Table 1). The EO composition of *S. kitaibelii* varied considerably in the samples from Serbia [20]. The cited authors found differences between the populations of *S. kitaibelii* in terms of the EO composition and the presence of chemotypes, namely geraniol, *p*-cymene, and limonene, with a high abundance of linalool and borneol [7,20]. Additionally, a prediction model, based on retention indices of *S. kitaibelii* EO, was developed [21]. 

According to the Convention on Biological Diversity (CBD), a Global Strategy for Plant Conservation [22] was approved in 2002. The protection of plant resources was the main objective of this strategy through the development of approaches for *in situ* and *ex situ* conservation of plants [22]. The introduction of wild species into cultivation is a way to limit their collection, and such measures will preserve their populations instead of destroying them. In order to introduce *S. pilosa* and *S. kitaibelii* as new cultivated crops, it is necessary to identify clones with high EO concentrations and desirable compositions. 

The objectives of this study were to: (1) establish the intrapopulation diversity and variability of *S. pilosa* EO content and composition in Bulgaria; (2) assess the interpopulation variability of *S. kitaibelii* EO (EO) content and composition and compare the EO composition of *S. kitaibelii* with that of a typical *S. montana;* and (3) identify prospective populations to be used for further selection and breeding of the two species. The working hypothesis was that the EO content and composition of *S. pilosa* at the intrapopulation level and *S. kitaibelii* in different populations will differ significantly.

## 2. Results and Discussion

### 2.1. Total EO Content (Yield)

#### 2.1.1. *Satureja pilosa*

The multiple means comparison results shown in Table 2 reveal the presence of considerable variability of the EO yield between samples at the intrapopulation level (each plant is one sample, a total of 12 plants). In the first population from the Balkan Mountains (the village of Selce), the oil yield between the different plants (samples) varied from 1.27% to 2.15%, while in the second population (the village of Samokitka in the Rhodope Mountains), the EO yield varied from 0.54% to 1.89%. In some of the studied samples the amount of EO was high (1.89–2.15%), and the results obtained by us were close to the yield obtained by Bezić et al. [26] for other types of *Satureja* (*S. montana* L. 2.8%, *S. cuneifolia* Ten. 2.6%, *S. subspicata* Vis. 2.0%, *S. visianii* Šilić 2.4%). Similar variability in the *S. pilosa* EO yield was detected in previous research of the populations in the territory of Bulgaria [12,17], Turkey [14], and Greece [13]. Overall, the data of the *S. pilosa* EO yield on the intrapopulation level cannot be compared with literature data because our study reported the yield of 24 individual plants (samples) within two geographically isolated populations. Very often, researchers take and analyze a compound sample of a given population. Mostly, genetic features affect the yield of *S. pilosa* because: (1) the samples were collected at the same time; (2) the samples were at the same phenological stage; (3) the plants within a population were growing under the same climatic conditions; and (4) the EO was extracted using the same method. Generally, the EO yield of *S. pilosa* varied from 0.54% to 2.15% between the two populations (Table 2).

#### 2.1.2. *Satureja kitaibelii*

The *S. kitaibelii* EO yield from eleven locations varied from 0.04% (Kostenkovci 1) to 0.43% (Gradec 1), while the EO yield of cultivated *S. montana* was 0.70% (Table 3). A similar variation of EO yield of *S. kitaibelii* was previously reported in one population each in Bulgaria (0.19%) [17] and Serbia (0.11% to 0.27%) [20].

### 2.2. Qualitative Composition of Essential Oil (EO)

#### 2.2.1. *Satureja pilosa*

Overall, monoterpenes was the main class of *S. pilosa* EO (93.96% to 98.61%) at the intrapopulation level (Table 2). The same results were found in a previous study on *S. pilosa* samples collected from 33 locations [12]. The most abundant compounds of class monoterpenes were thymol, carvacrol, *p*-cymene, and *γ*-terpinene (Supplementary Table S1). 

The analysis of variance results shown in Table 4, followed by the multiple means comparison results shown in Table 5 and Table 6, reveal that the EO profile of *S. pilosa* was specific to each population studied, and the EO had a clearly differentiated profile, which supports our working hypothesis. For example, carvacrol (38.99–77.09%) was the main compound of EO in 10 samples collected from the Balkan Mountains (village of Selce), and thymol (19.34% and 1.55%, respectively) was found in only two samples from this population (Table 5). For the samples from the Eastern Rhodope Mountains (Samokitka) in the EO composition, thymol predominated (52.52–72.20%), and in none of the samples was the presence of carvacrol detected. Bezić et al. [26] showed that the production of phenolic compounds in the EO was stimulated by hot and dry conditions of the environment. The studied populations of *S. pilosa* were found in petrophytic cenoses of the xerophytic mountain range. The plants were located on open, rocky terrain with southern exposure and high solar radiation, which only partly supports the assumption of Bezić et al. [26]. In this study, the population in the Balkan Mountains (Selce) was under the influence of a temperate continental climate zone and lower temperatures, while the population in the Eastern Rhodope Mountains (Samokitka) was under the influence of a Mediterranean climate characterized by higher temperatures [27]. Additionally, Bezić et al. [26] showed that the yield of carvacrol varied during ontogenesis and peaked during blossoming and high summer temperatures. In this study, the results from the population in the Eastern Rhodope Mountains (Samokitka) do not support the report of Bezic et al. [26]; the plants were grown at higher temperatures but carvacrol was not identified in them. Therefore, one may assume that temperature during vegetation may be less significant on carvacrol accumulation compared with that of plant genetics. Apparently, the EO profile of *S. pilosa* is a function of mostly genetic traits. 

A total of 18 compounds were identified in the EO of the species from both studied populations, as *p*-cymene (9.53–24.54%), *γ*-terpinene (1.23–10.75%), and terpinen-4-ol (0.36–3.87%) were present in all analyzed samples (Table 4, Table 5, Table 6 and Table 7). This is not surprising, because *p*-cymene and *γ*-terpinene are precursors in the biosynthesis of thymol and carvacrol [28]. Poulose and Croteau [28] noted that thymol is biosynthesized by the aromatization of *γ*-terpinene to *p*-cymene followed by hydroxylation of *p*-cymene. Thymol and carvacrol have important biological activities such as antibacterial activity, antioxidant activity, anticancer potency, and antiseptic activity [29,30,31]. Previous studies reported thymol, carvacrol, *γ*-terpinene, and *p*-cymene as the main compounds of *Satureja* species EO [13,32,33,34,35]. Furthermore, the cited authors reported quite a few chemotypes in *Satureja* species [36,37,38]. Apparently, there is high variability of EO in *Satureja* species, and chemotypes are common in the genus *Satureja*. 

Sesquiterpenes was the second established class in the samples from both studied populations in minimal quantities (0.89–5.34%) (Table 3). From these, *trans*-caryophyllene and caryophyllene oxide were present in the EO of all analyzed samples (Table 6). Overall, this is the first comprehensive study on the endemic plant *S. pilosa* that assessed the variability of EO on intrapopulation level. Based on the % ratio of the major compounds of the monoterpenes that this intrapopulation study, six chemotypes from two populations of *S. pilosa* were identified. In the two studied populations, three different chemical groups emerged. For a population from the Balkan Mountains (village of Selce), the following distribution was found: in seven samples there was predominance of (1) the carvacrol, *p*-cymene type; in four samples (2) the carvacrol, p-cymene and γ-terpinene type; and in one sample (3) the carvacrol, *p*-cymene, thymol, and *γ*-terpinene type, respectively. EO chemical differentiation was also found in the samples collected from the Eastern Rhodope Mountains (Samokitka). In three of the analyzed samples from this population, the main EO compound groups were found to be (4) the *p*-cymene and thymol type; in one sample (5) the *p*-cymene, thymol, cis-*β*-ocimene, and *γ*-terpinene type; and in eight samples, the main EO compounds were (6) *p*-cymene, thymol, and *γ*-terpinene. The lowest values (lowest percent composition limits) for the respective compounds in the above groups were: 19.34% for thymol, 9.53% for *p*-cymene, 5% for *γ*-terpinene, and 38.99% for carvacrol. Similar variability in the EO content of *S. pilosa* was found in our previous study in 33 locations in Bulgaria, where we established five different chemotypes [12]. Similar intrapopulation and interpopulation variability in EO content and composition were observed on *S. rechingeri* Jamzad from populations in Iran [33]. We should note that in a previous study we found thymol and carvacrol in samples both from the Balkan Mountains and from the Eastern Rhodope Mountains [12]. Both of the studied populations are geographically isolated (allopatric), and there is a small possibility of genetic exchange between them. Due to the fact that *S. pilosa* is a cross-pollinated plant, genetic material is exchanged within populations. As a result of the genetic drift within populations, genes are combined with large effects and tight control of the biosynthesis [39]. Apparently, distinct patterns in compositional variability of EO were the result of a combination of genes; as a result, the *S. pilosa* EO composition is quite variable [12]. Due to ongoing genetic processes in the populations of *S. pilosa*, morphological differences in the species have been found [4,13]. It is on the basis of morphological differences that some authors have proposed new taxonomic ranks for *S. pilosa*. Anchev [4] proposed under rank *S. pilosa* subsp. *pilosa*, *S. pilosa* var. *slavjankae* Anchev for the territory of Bulgaria, while Dardioti et al. [13] proposed under rank *S. pilosa* subsp. *origanita* Dardioti & Kokkini for the territory of Greece. Obviously, populations of *S. pilosa* are not genetically balanced, and there are ecotypes of the species. 

In general, in order to select plants of the EO species rich in thymol, it is necessary to collect material from the population of the Eastern Rhodope Mountains (Samokitka) and carvacrol from the population of the Balkan Mountains (village of Selce).

#### 2.2.2. *Satureja kitaibelii*

**Figure 1:**
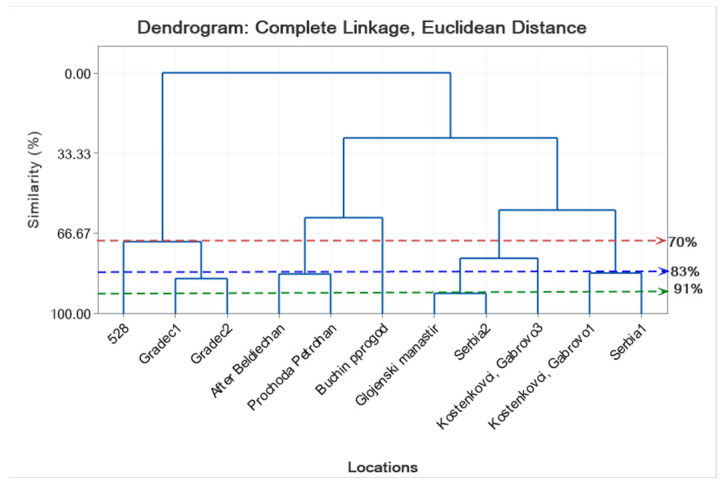
Complete linkage dendrogram showing the similarity of 11 locations in terms of *p*-cymene, *endo*-borneol, *trans*-caryophyllene, spathulenol, and caryophyllene oxide of *S. kitaibelii* and *S. montana* (number 528).

**Figure 2:**
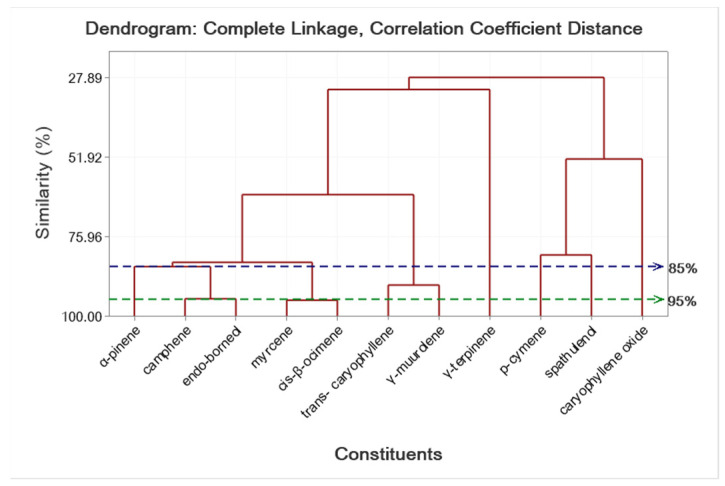
Complete linkage dendrogram showing the similarity of 11 constituents collected from 12 locations of *S. kitaibelii* and *S. montana* listed in Table 8.

The results of the EO composition of *S. kitaibelii* spread in nine Bulgarian locations and two Serbian locations very close to the Bulgarian border are shown in Table 8 and Table 9, as well as in Supplementary Table S2. The main class of compounds in 10 samples of the 11 studied samples of *S. kitaibelii* was the monoterpenes (39.40–81.23%), with the exception of one sample from Serbia, where monoterpenes were 17.75% and the unknown compounds reached 76.28%. For *S. montana* ssp. *montana* (cultivated), the amount of monoterpenes reached 92.87% (Table 8). In our study, we identified 33 substances in EO compositions that varied from sample to sample. EO compounds of the monoterpene class (39.40–81.23%) were *α*-pinene, *p*-cymene, *γ*-muurolene, *endo*-borneol, terpinen-4-ol, thymoquinone, geraniol, geranial, bornyl acetate, carvacrol, and geranyl acetate. Cluster analysis results of *S. kitaibelii* and *S. montana* ssp. *montana* that showed the similarity of location (11 levels) and the concentrations of 11 compounds are shown in Figure 1 and Figure 2. EO gas chromatographic analysis showed that samples collected from the same location have different EO compositions, although the plants developed under the same climatic conditions. For example, samples collected from the same location (Gradec 1 and Gradec 2) contained *p*-cymene amounts of 20.38% and 19.39%, respectively, and *γ*-muurolene amounts of 7% and 11.34%, respectively (Table 9). However, we should also note that in the first sample from this location (Gradec 1), terpinen-4-ol (14.95%) and bornyl acetate (5.77%) were found, which are compounds not present in the EO composition of the other sample from this location (Gradec 2). Variability in the EO composition of species has also been found for samples from other habitats. For example, the main EO compounds of the plants from Buchin prohod were *endo*-borneol (12.20%), *p*-cymene (5.56%), and *γ*-muurolene (18.72%), as well as carvacrol (5.06%) (Supplementary Table S2); while plant samples from Glozhene monastery contained geraniol (34.78%) and geranyl acetate (18.5%); and plants from Beldie han contained *endo*-borneol (11.98%), *p*-cymene (12.10%), *γ*-muurolene (6.68%), and *α*-pinene (4.78%) as major constituents (Table 8). In a previous study of samples from the same location in Bulgaria (Beldie han), Konakchiev and Tsankova [17] found high levels of limonene (15.7%) and *p*-cymene (13.1%). In this study, limonene was not found as a major constituent of the EO in plants from this population at Beldie han. Moreover, we did not find limonene in any of our analyzed samples. 

It is evident that the EO of *S. kitaibelii* varies considerably both between populations and within the populations themselves. A similar conclusion was reached by authors who analyzed samples of the species in Serbia [15,20,24]. The cited authors showed that there is EO variability both between different plant parts and between different populations of the species in Serbia [15,20,24]. Samples tested in Serbia showed high concentrations of geranyl acetate, geraniol, linalool, 1,8-cineole, limonene, bornyl acetate, and camphene [15,19,25]. 

The habitats of *S. kitaibelii* in the populations of Gradec, Beldie han, Buchin prochod, and Petrohan prochod are located in the western part of the Balkan Mountains in Bulgaria. The plants grow on limestone-based rocks in the north Bulgarian climate area under the influence of a temperate–continental climate zone [27]. According to Kopralev et al. [27], the Balkan Mountain range is divided into three parts—Northern, Central, and Eastern Balkan Mountains. The samples from the Western Balkan Mountains (locations of Gradec, Beldie han, Buchin prochod, and Petrohan prochod) have different phytochemical EO profiles than the samples obtained from the Central Balkan Mountains (Kostenkovci) and the samples from the locations that border the Western and Central Balkan Mountains (Glozhene monastery) (geraniol, geranyl acetate) (Table 7). For example, the samples from the Western Balkan Mountains are predominated by *p*-cymene, *γ*-muurolene, *endo*-borneol, bornyl acetate, and carvacrol, while thymoquinone, geranial, geranyl acetate, spathulenol, and caryophyllene oxide were the main compounds of samples from the Central Balkan Mountains (Kostenkovci). Different EO compositions (geraniol, geranyl acetate) were found in the samples from the locations that border the Western and Central Balkan Mountains (Glozhene monastery) (Table 7). 

Geraniol and geranyl acetate were found only in two samples from the Glozhene monastery (34.78% and 18.15%, respectively) and Kostenkovci (39.27% and 33.04%, respectively), while one of the samples from Serbia contained high concentrations only of geraniol (47.54%). The location of *S. kitaibelii* was spread around the Glozhene monastery and Kostenkovci (Central Balkan Mountains), and those from Serbia were geographically separated (allopatric). As is well-known, the Balkan Mountains comprise the longest mountain range in Bulgaria and Serbia [27]. Our studied locations of *S. kitaibelii* in the Balkan Mountains (Bulgaria and Serbia) represent the general geographical population of the species. Currently, *S. kitaibelii* populations are fragmented with a mosaic structure. Probably in the past, the studied locations in the geographical population were interconnected. The species had much larger areas of distribution and numbers, which is why there was a free exchange of genetic materials. This is probably one of the reasons why samples that are geographically separated have similar EO phytochemical compositions. Likely, the geographical location of the population of *S. kitaibelii* does not significantly affect the composition of the essential oil, but it is mainly genetic features that do so. 

In general, we can note that of the studied locations of *S. kitaibelii* with the most specific and different EO compositions were the samples collected from the Central Balkan Mountains (Kostenkovci). The main EO compounds for these samples (Kostenkovci) were thymoquinone (31.65%), geranial (5.68%), geranyl acetate (28.87%), spathulenol (6.09%), and caryophyllene oxide (8.61%) (Supplementary Table S2). In EO compositions of another sample from the same location were predominant thymoquinone (29.78%), geranyl acetate (30.73%), and *γ*-muurolene (7.25%) (Supplementary Table S2). We should emphasize that high concentrations of thymoquinone (31.65% and 29.8%, respectively) in the EO of *S. kitaibelii* were reported for the first time. Thymoquinone is one of the abundant compounds in *Nigella sativa* L. oil and is responsible for many pharmacological activities (inflammatory) and degenerative diseases, including cancer [40]. Geraniol is a commercially important compound and is very important for the flavor and fragrance industries [41]. Geraniol has a wide range of biological activities as an antimicrobial, antioxidant, and anti-inflammatory, but also as a chemoprevention agent for cancer [41]. The EO content of *S. kitaibelii* plants in the Kostenkovci population is of industrial interest, and plants can be selected for cultivation. 

In general, the results obtained in this study show that the main compounds of EO of *S. kitaibelii* were *p*-cymene, found in larger quantities in seven samples (>5%); *γ*-muurolene, in six samples; geraniol, in three samples; geranyl acetate, in four samples; caryophyllene oxide, in three samples; and thymoquinone, in two samples. Cluster analyses showed the similarity levels of locations and compounds of *S. kitaibelii* (Figure 1 and Figure 2). According to the statistical analysis results and the % ratio of the main EO compounds of *S. kitaibelii*, our studied samples can be grouped into 10 chemical groups as follows: (1) *p*-cymene, terpinen-4-ol, bornyl acetate, *γ*-muurolene, and *endo*-borneol type; (2) *p*-cymene, *cis*-*β*-ocimene, *trans*-*β*-ocimene, *endo*-borneol, carvacrol, and *γ*-muurolene type; (3) *α*-pinene, *p*-cymene, *cis*-*β*-ocimene, *trans*-*β*-ocimene, *endo*-borneol, and *γ*-muurolene type; (4) *p*-cymene, *cis*-*β*-ocimene, *endo*-borneol, *trans*-caryophyllene, and *γ*-muurolene type; (5) *p*-cymene, *endo*-borneol, carvacrol, and *γ*-muurolene type; (6) thymoquinone, geranial, geranyl acetate, spathulenol, and caryophyllene oxide type; (7) geraniol, geranyl acetate, and caryophyllene oxide type; (8) thymoquinone, geranyl acetate, and *γ*-muurolene type; (9) *p*-cymene, terpinen-4-ol, geraniol, and geranyl acetate type; and (10) geraniol, geranial, and caryophyllene oxide type. The lowest value (lowest percent composition limit) for the main, abundant EO compounds in the above groups was 5%. For the first time in Bulgaria, there were established EO chemical groups of the species. According to the literature data [7,15,19,20,21,25] on *S. kitaibelii*, EO showed several chemotypes from Serbia, namely: (1) *p*-cymene chemo type; (2) *p*-cymene and linalool chemo type; (3) geraniol chemo type, and (4) geraniol and borneol chemo type. 

Most often, morphological characteristics such as leaf shape, cup shape, tooth length, hairiness, etc., are the main metric indicators for the taxonomic recognition of species in *Satureja* species [3,4]. According to Flora of Europaea, the *S. montana* group includes six species, and *S. kitaibelii* was put as *S. montana* subsp. *kitaibelii* [3]. The taxonomic characteristics of *S. montana* are not very clear but are confusing taxonomically and therefore from a chorological point of view [19]. Generally, phytochemical characteristics of plants are additional criteria in the taxonomy, which is why we decided to compare the EO compositions of *S. montana* and *S. kitaibelii*. In general, the main EO compounds in *S. montana* ssp. *montana* (cultivated) were found to be *p*-cymene (21.32%), thymol (20.18%), and carvacrol (42.61%), and the EO profile of the species was closer to *S. pilosa* than to *S. kitaibelii*. From the results obtained in this study, the EO content of *S. kitaibelii* occupies an isolated position compared to *S. montana*. Only *p*-cymene, which was found in all samples of *S. kitaibelii* in only two samples—Gradec 1 (20.38%) and Gradec 2 (19.39%)—was in high concentrations close to those of *S. montana* (Table 9). Our results show a clear phytochemical differentiation of both species (*S. kitaibelii, S. montana*) and they confirm the results of other authors who compared EO of both species in Serbia [19,23].

## 3. Materials and Methods

### 3.1. Materials

The samples of *S. pilosa* Velen. and *S. kitaibelii* Wierzb. ex Heuff. (*S. montana* subsp. *kitaibelii)* were collected 20–30 July 2019, at the full flowering stage. The two *Satureja* species were identified on the basis of taxonomic features, according to the Handbook for Identification of Vascular Plants in Bulgaria [42]. The studied locations of *S. pilosa* and *S. kitaibelii* with the exact coordinates and altitude are shown in Supplementary Table S3. Voucher specimens of target species were deposited at the Herbarium of the Agricultural University, Plovdiv, Bulgaria (SOA) [43]. 

#### 3.1.1. *S. kitaibelii*

The materials used in this study were randomly selected from aboveground plant parts of *S. kitaibelii* (*S. montana* subsp. *kitaibelii)* populations in Bulgaria and in Serbia. Samples of *S. kitaibelii* were collected at nine locations across Bulgaria and two locations from Serbia, close to the Bulgarian border. The composition of the *S. kitaibelii* EO was compared with a typical species *S. montana* ssp. *montana* EO cultivated in the Institute of Field and Vegetable Crops Novi Sad, Department of Vegetable and Alternative Crops in Backi Petrovac, Serbia. Voucher specimens were confirmed and deposited at the Herbarium of the Faculty of Natural Science, University of Novi Sad, Serbia (BUNS), under number 2-1561.

#### 3.1.2. *S. pilosa*

The samples of the intrapopulation level of *S. pilosa* were collected from two populations in Bulgaria: (1) the Balkan Mountains (Selce), and (2) near the village of Samokitka in the East Rhodope Mountains. The materials of *S. pilosa* were collected from twelve individual plants per population. Each individual sample of *S. pilosa* was collected separately, and the plant was marked so that we can collect seeds in the future. 

### 3.2. Drying of the Samples

All *Satureja* samples were air dried in a shady area at temperatures below 30 °C to avoid oil losses and changes in the EO profile prior to the extraction.

### 3.3. Preparation of Samples for the EO Isolation

Subsamples of *S. kitaibelii* were generated randomly from each air-dried sample. The whole aboveground plant parts were used in this study. Each of the twelve collected plants per population (total 24) of *S. pilosa* represented an individual sample.

### 3.4. Essential Oil (EO) Isolation of the Satureja Biomass Samples

The EO of the aboveground plant parts was extracted via hydrodistillation in 2 L distillation units (Laborbio Ltd. Sofia, Bulgaria, www.laborbio.com, accessed on 1 May 2022) at the Research Institute for Roses and Medicinal Plants in Kazanluk, Bulgaria. Each extraction was performed in two replicates. Samples of dried aboveground plant parts (Supplementary Table S3) plus 0.800 L of water were placed in a Clevenger distillation system. After isolation of each subsample, the EO volume and weight were measured, and the EO samples were stored in a freezer until analyses. Here, we report the oil content (yield) based on weight in dried matter.

### 3.5. Gas Chromatography Mass Spectrometry Flame Ionization Detection (GC–MS–FID) Essential Oil Analysis

The extracted EO from all *Satureja* samples in two replications were analyzed for chemical profile by gas chromatography (GC)—mass spectroscopy (MS)—flame ionization detection (FID) techniques. Using a micropipette, 50 μL of oil (weight measured on a tared balance) from each sample was transferred into a 10 mL volumetric flask. Samples were brought to volume with CHCl_3_. A 1 mL aliquot of each diluted oil sample was placed by glass pipet into a GC vial for analysis. 

Oil samples were analyzed by GC–MS–FID on an Agilent (Santa Clara, CA, USA) 7890A GC system coupled to an Agilent 5975C inert XL MSD. Chemical standards and oils were analyzed using a DB-5 column (30 m × 0.25 mm fused silica capillary column, film thickness of 0.25 µm) operated using an injector temp of 240 °C, column temperature of 60 to 240 °C at 3 °C/min and held at 240 °C for 5 min, helium as the carrier gas, an injection volume of 1 µL (split ratio 25:1), and an MS mass range from 50 to 550. FID temperature was 300 °C. Post-column splitting was performed so that 50% of outlet flow proceeded to FID and 50% to mass spectrometry (MS) detection. 

Compounds were identified by Retention Index and Kovats Index analyses [44], and direct comparison of MS and retention time to authentic standards, and comparison of mass spectra with those reported in the NIST mass spectra database except for carvacrol methyl ether, as no standard was available. Commercial standards of α-thujene, *α*-pinene, camphene, oct-1-en-3-ol, myrcene, *α*-terpinene, *p*-cymene, *cis*-*β*-ocimene, *γ*-terpinene, terpinen-4-ol, thymoquinone, thymol, carvacrol, *trans*-caryophyllene, *β*-bisabolene, and caryophyllene oxide were obtained from Sigma-Aldrich (St. Louis, MO, USA). 

Compounds were quantified by performing area percentage calculations based on the total combined FID area. For example, the area for each reported peak was divided by total integrated area from the FID chromatogram from all reported peaks and multiplied by 100 to arrive at a percentage. The percentage of a peak is a percentage relative to all other constituents integrated in the FID chromatogram.

### 3.6. Statistical Analyses

#### 3.6.1. *Satureja pilosa*

The effect of location (Selce and Samokitka) and sample (695–697 and 728–736 in Selce, 715–727 in Samokitka) nested in location on 15 constituents (*α*-pinene, *α*-thujene, camphene, myrcene, *p*-cymene, *α*-terpinene, *γ*-terpinene, unknown, terpinen-4-ol, *trans*-caryophyllene, caryophyllene oxide, *cis*-*β*-ocimene, thymoquinone, thymol, and carvacrol) was determined using a nested design with the two effects in the model being location and sample (location). The analysis of variance (ANOVA) was done using Proc Mixed of SAS [45]. Since there were 12 samples from the Selce location and 12 samples from the Samokitka location, the multiple means comparison of the samples nested within location was done on 24 means, which is large. Therefore, to minimize the potential over inflation of Type II experiment-wise error rate, LSD at 1% significance level was used for letter grouping. 

For each response variable, normal distribution and constant variance assumptions on the error terms were validated as described in Montgomery [46], and an appropriate transformation was applied on some of the response variables where the assumption was violated; however, the results in the tables are presented after back transforming them to the original scale. 

#### 3.6.2. *Satureja kitaibelii* and *S. montana* (Cultivated)

The significance of the location (11 levels) effect on EO yield, and the concentrations of 11 constituents (*α*-pinene, camphene, myrcene, *p*-cymene, *cis*-*β*-ocimene, *γ*-terpinene, *endo*-borneol, *trans*-caryophyllene, *γ*-muurolene, spathulenol, and caryophyllene oxide) were determined using ANOVA, and the data were analyzed using Proc Mixed of SAS. Model assumptions were also verified as described in Montgomery [46]. Since location had significant (*p*-value < 0.05) effects on EO yield and the concentrations of all constituents, Tukey’s multiple range test at α = 5% was used to generate letter groupings. 

To determine the similarity level of the locations in terms of all 11 constituents, and the constituents in terms of all 11 locations, cluster analysis (complete linkage clustering) was conducted to generate dendrograms for the locations and the constituents as described in Johnson and Wichern [47]. 

## 4. Conclusions

This is the first comprehensive study on the EO chemical variability of the Balkan endemic *S. pilosa* on the intra-population level and the variability of EO of *S. kitaibelii* collected from wild populations in Bulgaria. The quantitative and qualitative EO compositions of *S. pilosa* in both of the studied populations varied widely. Expression of the *S. pilosa* EO variations are the established chemical types that are most probably due to genetic specificity. In the first population (Balkan Mountains) *S. pilosa* chemical groups were: (1) the carvacrol and *p*-cymene type; (2) the carvacrol, *p*-cymene, and *γ*-terpinene type; and (3) the carvacrol, *p*-cymene, thymol, and *γ*-terpinene type; while for the second population (the Eastern Rhodope Mountains), the chemotypes included (1) the *p*-cymene and thymol type; (2) the *p*-cymene, thymol, *cis*-*β*-ocimene, and *γ*-terpinene type; and (3) the *p*-cymene, thymol, and *γ*-terpinene type. 

The interpopulation variability in the Bulgarian populations of *S. kitaibelii* EO was studied for the first time. Significant variability in EO quantity and quality was found. Thus, 10 chemical types were identified. The presence of thymoquinone in the EO of *S. kitaibelii* from the population in Kostenkovci is reported for the first time. If the chemical profile of the plant population at Kostenkovci is of industrial interest, then plants can be selected for introduction into a crop culture.

## Figures and Tables

**Table 1:** The main compounds of essential oils of this study and literature reports on *S. kitaibelii*.

References	The Main Compounds in % of *S. kitaibelii*	Country
Our data	*α*-pinene (0.00–4.91); thymoquinone (0.00–32.47); camphene (0.00–2.66); myrcene (0.00–1.42); *p*-cymene (1.47–20.38); *cis*-*β*-ocimene (0.00–7.42); *γ*-terpinene (0.00–4.56); *endo*-borneol (2.01–12.20); *trans*-caryophyllene (1.50–5.71); *γ*-muurolene (0.00–18.72); spathulenol (0.61–6.08); caryophyllene oxide (1.70–8.61); geraniol (0.00–54.55); carvacrol (0.00–10.11); thymol (0.00–29.65).	Bulgaria/Serbia
Dodoš et al. [23]	*p*-cymene (13.1–13.2); *trans*-caryophyllene (17.1–21.3); caryophyllene oxide (3.4); limonene (4.6).	Serbia
Aćimović et al. [7]	*α*-pinene (2.5); camphene (1.1); myrcene (0.8); *p*-cymene (24.4); *cis*-*β*-ocimene (2.3); *γ*-terpinene (0.9); *endo*-borneol (4.9); *trans*-caryophyllene (2.8); *γ*-muurolene (0.1); spathulenol (2.0); caryophyllene oxide (2.9); limonene (13.5); borneol (0.1); carvacrol (2.3).	Serbia
Aćimović et al. [21]	*α*-pinene (2.5); camphene (1.1); myrcene (0.8); *p*-cymene (24.4); *cis*-*β*-ocimene (2.3); *γ*-terpinene (0.9); *endo*-borneol (4.9); *trans*-caryophyllene (2.8); *γ*-muurolene (0.1); spathulenol (2.0); caryophyllene oxide (2.9); limonene (13.5); borneol (0.1); carvacrol (2.3).	Serbia
Dodoš et al. [15]	*α*-pinene (2.0–3.2); camphene (1.4–2.2); myrcene (0.2–0.4); *p*-cymene (5.5–21.9); *cis*-*β*-ocimene (0.1–0.5); *γ*-terpinene (0.4–1.0); *trans*-caryophyllene (1.2–4.2); *γ*-muurolene (0.0–0.5); spathulenol (1.2–5.8); caryophyllene oxide (1.6–5.9); limonene (5.2–8.0); geraniol (0.0–12.0); borneol (7.6–7.7); carvacrol (0.4–0.6).	Serbia
Đorđević et al. [20]	*α*-pinene (06–1.1); camphene (0.3); myrcene (0.2–0.4); *p*-cymene (1.4–4.3); *γ*-terpinene (0.2–0.6); *trans*-caryophyllene (2.3–3.3); *γ*-muurolene (0.1–0.2); spathulenol (1.5–2.8); caryophyllene oxide (4.4–5.2); limonene (4.3–7.9); geraniol (24.0–30.03); borneol (1.8–3.7).	Serbia
Mihajilov-Krstev et al. [24]	*α*-pinene (1.8); camphene (0.6); myrcene (0.6); *p*-cymene (10.0); *cis*-*β*-ocimene (1.9); *γ*-terpinene (1.6); spathulenol (2.9); caryophyllene oxide (5.9); limonene (3.8); geraniol (0.9); borneol (8.9).	Serbia
Kundaković et al., 2011, [18]	*α*-pinene (2.2–6.3); camphene (0.0–2.0); myrcene (0.2–1.7); *p*-cymene (14.7–27.9); *cis*-*β*-ocimene (0.3–4.5); *γ*-terpinene (1.7–11.6); *endo*-borneol (5.0–9.9); spathulenol (0.5–4.0); caryophyllene oxide (0.9–2.4); limonene (8.8–13.6); carvacrol (0.5–7.1).	Serbia
Konakchiev and Tsankova, [17]	*α*-pinene (5.5); camphene (3.2); myrcene (1.8); *p*-cymene (13.1); *cis*-*β*-ocimene (4.8); *γ*-terpinene (3.1); *γ*-muurolene (0.1); spathulenol (0.6); caryophyllene oxide (0.8); limonene (15.7); borneol (2.2); carvacrol (2.0).	Bulgaria
Slavkovska et al. [19]	*α*-pinene (6.0); camphene (3.7); myrcene (1.0); *p*-cymene (20.9); *γ*-terpinene (2.0); limonene (16.0); borneol (9.8); carvacrol (0.9).	Yugoslavia
Chalchat et al. [25]	*α*-pinene (1.1); camphene (0.5); myrcene (1.2); *p*-cymene (33.6); *cis*-*β*-ocimene (0.3); spathulenol (2.1); caryophyllene oxide (2.3); borneol (3.6); carvacrol (14.1).	Serbia

**Table 2:** Mean (%) oil yield of Bulgarian *S. pilosa* monoterpenes, sesquiterpenes, and an unknown group obtained from 24 samples collected at the populations of the Balkan Mountains (Selce) and the East Rhodope Mountains (Samokitka).

№ Sample (Location)	Oil Yield	Monoterpenes	Sesquiterpenes	Unknown Group
695 (Sel)	1.27 bcdefg	93.96 j	5.61 a	0.46 bcd
696 (Sel)	1.98 ab	96.96 ghi	2.77 bcd	0.29 cd
697 (Sel)	1.57 abcde	97.27 efghi	2.55 bcde	0.19 d
728 (Sel)	2.15 a	97.34 defghi	1.47 ghijk	1.19 a
729 (Sel)	1.7 abc	94.11 j	5.34 a	0.45 bcd
730 (Sel)	1.60 abcd	98.03 abcde	1.41 hijk	0.42 bcd
731 (Sel)	1.94 ab	97.19 fghi	2.48 bcde	0.33 bcd
732 (Sel)	1.67 abcd	96.69 hi	2.10 defgh	0.55 bc
733 (Sel)	1.37 bcdef	98.01 abcdef	1.61 fghijk	0.39 bcd
734 (Sel)	1.44 abcdef	94.17 j	1.63 fghij	0.55 bc
735 (Sel)	1.66 abcd	96.70 hi	2.86 bc	0.45 bcd
736 (Sel)	1.61 abcd	97.28 efghi	2.25 cdef	0.47 bcd
715 (Sam)	0.82 fg	98.34 ab	1.31 ijk	0.37 bcd
717 (Sam)	0.54 g	97.21 efghi	2.47 bcde	0.34 bcd
718 (Sam)	0.96 defg	98.14 abcd	1.39 hijk	0.47 bcd
719 (Sam)	1.35 bcdef	98.36 ab	1.48 ghijk	0.31 bcd
720 (Sam)	1.78 abc	98.30 abc	1.15 jk	0.54 bcd
721 (Sam)	0.84 efg	96.58 i	3.00 b	0.33 bcd
722 (Sam)	0.84 efg	97.50 cdefgh	2.18 cdefg	0.32 bcd
723 (Sam)	0.85 efg	97.70 bcdefg	1.92 efghi	0.54 bcd
724 (Sam)	1.71 abc	98.61 a	0.91 k	0.49 bcd
725 (Sam)	1.49 abcdef	98.33 abc	1.18 jk	0.51 bcd
726 (Sam)	1.18 cdefg	98.47 ab	0.89 k	0.65 b
727 (Sam)	1.89 abc	97.75 bcdefg	1.59 fghijk	0.33 bcd

Selce—Sel; Samokitka—Sam. Within each column, means sharing the same letter are not significantly different;

**Table 3:** Mean EO yield (%) of *S. kitaibelii* collected at 11 locations.

Location	EO Yield	Location	EO Yield
After Beldie han	0.23 b	Kostenkovci 1	0.04 b
Buchin prohod	0.15 b	Kostenkovci 2	0.11 b
Glojenski manastir	0.15 b	Kostenkovci 3	0.28 b
Gradec1	0.43 ab	Petrohan Prochod	0.16 b
Gradec2	0.35 ab	Serbia1	0.09 b
Serbia 2	0.08 b	*S. montana* spp. *montana* Cultivated	0.70 a

Within each column, means sharing the same letter are not significantly different.

**Table 4:** ANOVA *p*-values that show the significance of the effects of location and samples (location) on 15 constituents of *S. pilosa*. * = no data from Samokitka.

Source of Variation	*α*-Thujene	*α*-Pinene	Camphene	Myrcene	*α*-Terpinene
Location	0.001	0.001	0.001	0.001	0.001
Sample (Location)	0.003	0.001	0.001	0.001	0.001
	***p*-cymene**	***γ*-terpinene**	**unknown**	**terpinen-4-ol**	***trans*-caryophyllene**
Location	0.001	0.001	0.214	0.001	0.001
Sample (Location)	0.001	0.001	0.001	0.001	0.001
	**caryophyllene oxide**	***cis*-*β*-ocimene**	**thymoquinone**	**thymol**	**carvacrol**
Location	0.001	0.001	0.428	0.001	*
Sample (Location)	0.001	0.001	0.873	0.001	0.001

**Table 5:** Mean concentration (%) of *α*-thujene, *α*-pinene, camphene, myrcene, *α*-terpinene, thymol, carvacrol, and *p*-cymene of *S. pilosa* obtained from 24 samples collected at the 2 locations (Sel = Selce, Sam = Samokitka).

Sample (Location)	*α*-Thujene	*α*-Pinene	Camphene	Myrcene	*α*-Terpinene	Thymol	Carvacrol	*p*-Cymene
695 (Sel)	0.29 e	0.28 hi	0.21 hi	0.76 ghi	0.88 h	*	72.86 abc	12.71 hijk
696 (Sel)	0.41 de	0.50 defghi	1.85 a	0.87 fghi	1.31 efgh	*	74.03 abc	13.93 ghij
697 (Sel)	0.29 e	0.23 i	0.10 j	0.71 ghi	0.78 h	*	77.69 a	11.43 jk
728 (Sel)	1.4 a	0.92 abc	0.21 hi	3.02 a	2.57 a	19.34 h	38.99 f	19.55 bcde
729 (Sel)	0.56 cde	0.32 ghi	0.15 ij	0.60 hi	1.30 efgh	1.55 i	63.40 de	21.13 bcd
730 (Sel)	1.01 abc	0.55 cdefghi	0.15 ij	1.42 cde	1.58 cdef	*	68.88 cde	12.40 hijk
731 (Sel)	0.69 bcde	0.44 efghi	0.22 ghi	0.69 ghi	1.05 fgh	*	74.19 abc	15.00 efghij
732 (Sel)	0.91 abcd	0.63 cdefghi	0.28 fgh	0.43 i	1.05 fgh	*	69.08 cd	21.28 bcd
733 (Sel)	0.41 de	0.34 ghi	0.13 ij	0.62 hi	0.95 gh	*	74.78 abc	16.13 defghi
734 (Sel)	0.90 abcd	0.65 cdefghi	0.36 efg	1.02 defgh	1.35 defgh	*	62.06 e	22.95 b
735 (Sel)	0.60 cde	0.37 fghi	0.16 ij	0.93 efghi	0.92 gh	*	77.09 ab	9.53 k
736 (Sel)	0.68 bcde	0.47 efghi	0.19 hi	1.18 defg	1.19 efgh	*	70.47 bc	13.61 ghij
715 (Sam)	1.17 ab	0.92 abcd	0.47 de	2.13 b	2.00 abc	70.24 ab	*	12.34 ijk
717 (Sam)	1.12 abc	0.72 abcdefg	0.37 ef	2.14 b	1.65 bcde	52.52 g	*	24.54 b
718 (Sam)	1.11 abc	0.67 cdefgh	0.44 de	1.88 bc	2.18 ab	58.19 ef	*	18.90 bcdef
719 (Sam)	0.91 abcd	0.76 abcdef	0.96 b	1.78 bc	2.04 abc	69.39 abc	*	11.44 jk
720 (Sam)	0.58 cde	0.37 fghi	0.28 fgh	0.97 defgh	1.58 cdef	72.20 a	*	16.58 defgh
721 (Sam)	0.81 bcde	0.71 bcdefg	0.61 cd	1.38 cdef	1.72 bcde	62.60 de	*	20.38 bcd
722 (Sam)	1.11 abc	0.97 abc	1.01 b	1.84 bc	2.03 abc	59.75 e	*	17.37 cdefg
723 (Sam)	0.82 bcde	0.56 cdefghi	0.30 efgh	1.11 defgh	1.77 bcde	64.69 cd	*	22.00 bc
724 (Sam)	0.94 abcd	0.81 abcde	0.75 bc	1.49 cd	1.61 bcdef	68.87 abc	*	15.22 efghij
725 (Sam)	0.98 abcd	1.14 a	0.86 bc	1.03 defgh	2.17 ab	53.65 fg	*	31.91 a
726 (Sam)	0.97 abcd	0.89 abcd	0.92 b	1.04 defgh	1.47 cdefg	66.80 bcd	*	20.21 bcd
727 (Sam)	1.03 abc	1.11 ab	0.94 b	1.81 bc	1.89 bcd	66.98 bcd	*	14.50 fghij

* not detected; within each column, means sharing the same letter are not significantly different.

**Table 6:** Mean concentration (%) of thymoquinone, terpinen-4-ol, *trans*-caryophyllene, caryophyllene oxide, unknown, cis-*β*-ocimene, and *γ*-terpinene obtained from 24 samples of *S. pilosa* collected at the 2 locations (Sel = Selce, Sam = Samokitka).

Sample (Location)	Thymoquinone	Terpinen-4-ol	*Trans*-Caryophyllene	Caryophyllene Oxide	Unknown	*Cis*-*β*-Ocimene	*γ*-Terpinene
695 (Sel)	*	0.64 cdefgh*	3.73 a	1.88 a	0.46 bcd	1.47 cd	3.54 fghi
696 (Sel)	*	0.74 cdefg	2.17 b	0.59 defg	0.29 cd	*	4.64 cdefgh
697 (Sel)	0.29 a	0.43 gh	1.55 c	1.00 cd	0.20 d	1.23 ef	3.96 efghi
728 (Sel)	0.63 a	0.66 cdefgh	0.97 g	0.49 defg	1.18 a	1.09 ef	8.69 ab
729 (Sel)	0.53 a	0.50 fgh	3.73 a	1.61 ab	0.45 bcd	0.31 ij	3.36 ghi
730 (Sel)	0.28 a	0.36 h	0.99 fg	0.41 efg	0.41 bcd	0.39 ghi	10.75 a
731 (Sel)	0.49 a	0.59 cdefgh	1.58 c	0.90 cde	0.33 bcd	0.60 g	3.05 hi
732 (Sel)	0.55 a	0.71 cdefg	1.52 c	1.26 bc	0.54 bc	0.12 j	1.23 i
733 (Sel)	0.56 a	0.64 cdefgh	1.05 efg	0.56 defg	0.39 bcd	*	3.19 ghi
734 (Sel)	0.48 a	0.55 defgh	0.96 gh	0.67 defg	0.55 bc	0.45 ghi	6.72 bcde
735 (Sel)	0.42 a	0.51 efgh	1.98 b	0.88 cde	0.45 bcd	0.55 gh	5.3 cdefgh
736 (Sel)	0.40 a	0.60 cdefgh	1.56 c	0.69 def	0.47 bcd	0.51 ghi	7.08 bcd
715(Sam)	*	0.52 efgh	0.58 i	0.12 g	0.37 bcd	1.13 ef	7.04 bcd
717 (Sam)	*	0.75 cdefg	1.45 cd	1.02 cd	0.34 bcd	5.75 a	7.19 bc
718 (Sam)	*	0.73 cdefg	0.81 ghi	0.59 defg	0.46 bcd	2.97 b	10.65 a
719 (Sam)	*	0.64 cdefgh	1.13 defg	0.35 efg	0.31 cd	1.13 ef	8.92 ab
720 (Sam)	*	0.66 cdefgh	0.61 hi	0.54 defg	0.54 bcd	0.13 j	4.68 cdefgh
721 (Sam)	*	0.83 cde	1.33 cdef	1.67ab	0.32 bcd	1.04 f	6.15 bcdefg
722 (Sam)	*	0.76 cdef	1.39 cde	0.8 cdef	0.32 bcd	1.28 de	10.74 a
723 (Sam)	*	3.87 a	1.07 efg	0.853 cdef	0.54 bcd	0.54 gh	4.09 defghi
724 (Sam)	*	0.86 cd	0.61 i	0.30 fg	0.49 bcd	0.33 hij	7.28 bc
725 (Sam)	0.831 a	1.29 b	0.59 i	0.58 defg	0.50 bcd	0.42 ghi	2.89 hi
726 (Sam)	0.367 a	0.73 cdefg	0.48 i	0.41 efg	0.65 b	1.01 f	3.54 fghi
727 (Sam)	*	0.91 c	0.97 g	0.62 defg	0.33 bcd	1.66 c	6.52 bcdef

* not detected; within each column, means sharing the same letter are not significantly different.

**Table 7:** Mean concentration (%) of *α*-thujene, *α*-pinene, camphene, myrcene, *α*-terpinene, *p*-cymene, *γ*-terpinene, unknown, terpinen-4-ol, *trans*-caryophyllene, caryophyllene oxide, cis-*β*-ocimene, thymoquinone, thymol, and carvacrol of *S. pilosa* collected at the 2 locations.

**Location**	**α-thujene**	**α-pinene**	**camphene**	**myrcene**	**α-terpinene**
**Samokitka**	0.961 a*	0.803 a	0.617 a	1.549 a	1.843 a
**Selce**	0.678 b	0.473 b	0.245 a	1.020 b	1.244 b
	***p*-cymene**	***γ*-terpinene**	**unknown**	**terpinen-4-ol**	***trans*-caryophyllene**
**Samokitka**	18.20 a	6.641 a	0.475 a	1.045 a	0.918 b
**Selce**	15.36 b	5.126 b	0.430 a	0.577 b	1.815 a
	**caryophyllene oxide**	***cis*-*β*-ocimene**	**thymoquinone**	**thymol**	**carvacrol**
**Samokitka**	0.653 b	1.449 a	0.599 a	63.82 a	*
**Selce**	0.911 a	0.671 b	0.463 a	10.44 b	68.63

* no detected; within each constituent, means sharing the same letter are not significantly different.

**Table 8:** Mean classes of monoterpenes, sesquiterpenes, and the unknown group obtained from 11 populations of *S. kitaibelii* and *S. montana* ssp. *montana* (cultivated).

Location/Class	Bh	Bp	Gm	G1	G2	K1	K2	K3	PP	S1	S2	Sm
Monoterpenes	57.31	39.40	71.91	72.3	57.24	74.36	81.23	74.06	47.13	68.19	17.75	92.87
Sesquiterpenes	16.98	30.08	12.23	9.75	22.39	21.77	16.61	21.4	27.915	21.75	5.55	5.52
Unknown	15.92	21.85	11.9	15.86	15.41	3.89	2.15	4.55	0.00	10.02	76.28	1.62

Beldie han—Bh; Buchin prohod—Bp; Glojenski manastir—Gm; Gradec1—G1; Gradec2—G2; Kostenkovci 1—K1; Kostenkovci 2—K2; Kostenkovci 3—K3; Prochoda Petrohan—PP; Serbia1—S1; Serbia2—S2; *S. montana* (cultivated)—Sm.

**Table 9:** Mean concentration (%) of *α*-pinene, camphene, myrcene, *p*-cymene, *cis*-*β*-ocimene, *γ*-terpinene, *endo*-borneol, *trans*-caryophyllene, *γ*-muurolene, spathulenol, and caryophyllene oxide of *S. kitaibelii* collected at 11 locations and *S. montana* (cultivated).

Location	*α*-Pinene	Camphene	Myrcene	*p*-Cymene	*Cis*-*β*-Ocimene	*γ*-Terpinene	*Endo*-Borneol	*Trans*-Caryophyllene	*γ*-Muurolene	Spathulenol	Caryophyllene Oxide
Bh	4.78 a *	2.66 a	1.27 a	12.10 abc	5.63 ab	0.61 b	11.98 a	3.15 bcd	6.68 b	3.63 bc	2.20 b
Bp	2.44 ab	1.81 abc	1.42 a	5.56 bcd	4.65 ab	0.66 b	12.20 a	4.76 ab	18.72 a	0.61 f	1.69 b
Gm	0.65 b	0.46 c	0.41 b	5.39 bcd	0.59 b	1.20 b	2.50 de	2.81 bcd	3.60 b	1.62 ef	3.07 b
G1	2.11 ab	1.53 abc	0.74 b	20.38 a	1.61 b	4.56 a	4.97 bc	2.29 cd	7.00 b	1.90 def	1.70 b
G2	2.94 ab	1.56 abc	1.28 a	19.39 a	7.42 a	0.95 b	5.53 b	4.29 abc	11.34 ab	3.41 bcd	3.35 b
K1	ND	ND	ND	1.47 d	ND	ND	2.54 de	3.12 bcd	7.26 b	6.08 a	8.61 a
K2	ND	ND	0.92 ab	1.05 e	ND	ND	0.73 e	4.90 abc	3.96 b	ND	7.75 a
K3	ND	ND	ND	1.64 cd	ND	ND	1.78 de	3.13 bcd	ND	1.48 ef	3.31 b
PP	4.91 a	2.06 ab	1.28 a	13.61 ab	5.51 ab	0.86 b	10.27 a	5.71 a	11.59 ab	2.84 cde	3.06 b
S1	1.44 ab	0.66 bc	ND	4.10 bcd	ND	0.72 b	3.07 cd	5.00 ab	2.06 b	4.73 ab	7.44 a
S2	2.38 ab	0.50 bc	0.65 b	6.34 bcd	0.62 b	0.65 b	2.01 de	1.50 d	1.36 b	0.98 f	2.60 b
Sm	0.74 b	0.19 c	0.43 b	21.32 a	ND	1.29 b	0.84 e	1.52 d	ND	1.12 f	1.22 b

Beldie han—Bh; Buchin prohod—Bp; Glojenski manastir—Gm; Gradec1—G1; Gradec2—G2; Kostenkovci 1—K1; Kostenkovci 2—K2; Kostenkovci 3—K3; Prochoda Petrohan—PP; Serbia1—S1; Serbia2—S2; *S. montana* (cultivated)—Sm; not detected—ND; *—within each column, means sharing the same letter are not significantly different.

## Data Availability

Data is contained within the article.

## Supplementary Materials

The following supporting information can be downloaded at: https://www.mdpi.com/article/10.3390/molecules27103153/s1; Table S1: Constituents and concentrations of *Satureja pilosa* samples (intrapopulation level) were collected from two populations in Bulgaria: (1) The Balkan Mountains (Selce), and (2) near the village of Samokitka (East Rhodopes); Table S2: Constituents and concentrations of *S. kitaibelii* (*S. montana* subsp. *kitaibelii)* populations in Bulgaria and Serbia. Table S3: Locality, coordinates and used samples in population of *Satureja pilosa* and *S. kitaibelii* in Bulgaria and Serbia.

## Sample Availability

Small samples of the some of the essential oils are available from the authors.
