# Supplementary material for: Phytochemical Variability of Essential Oils of Two Balkan Endemic Species: Satureja pilosa Velen. and S. kitaibelii Wierzb. ex Heuff. (Lamiaceae)
Source: Molecules. 2022 May 14;27(10):3153. doi: 10.3390/molecules27103153 (PMC9147943; doi:10.3390/molecules27103153)
Supplement: Supplementary file 1 [file molecules-27-03153-s001.zip › molecules-1715765-supplementary.pdf]

Phytochemical variability of essential oils of two Balkan en-demic species *Satureja pilosa* Velen. and *S. kitaibelii* Wierzb. ex Heuff. (Lamiaceae)

Table S1

Constituents and concentrations of *Satureja pilosa* samples (intrapopulation level) were collected from two populations in Bulgaria: (1) The Balkan Mountain (Selce) and (2) near the village of Samokitka (East Rhodopes).

**Number of constituents**	**Volatile constituents of *Satureja pilosa***	**RTime**	**Concentration Range % (min-max) Stara planina**	**Concentration Range % (min-max)** Izt. Rodopi
**1**	α-thujene	5.819	0.00-1.50	0.46-1.38
**2**	α-pinene	6.021	0.00-1.12	0.29-1.30
**3**	camphene	6.430	0.00-3.48	0.23-1.10
**4**	oct-1-en-3-ol	7.236	0.00-0.38	0.22-0.84
**5**	myrcene	7.601	0.00-3.13	0.83-2.36
**6**	α-terpinene	8.482	0.28-2.69	1.36-2.54
**7**	p-cymene	8.755	9.00-23.97	11.06-38.32
**8**	cis-β-ocimene	9.175	0.00-1.49	0.11-6.31
**9**	γ-terpinene	9.983	0.00-11.48	2.10-10.90
**10**	unknown	10.313	0.00-1.20	0.24-0.72
**11**	terpinen-4-ol	14.755	0.00-0.73	0.49-6.71
**12**	carvacrol methyl ether	17.538	0.00-0.58	0.00-1.04
**13**	thymoquinone	17.747	0.00-2.32	0.00-1.38
**14**	thymol	19.912	0.00-19.80	47.24-70.85
**15**	carvacrol	20.256	37.76-83.07	0.00-0.00
**16**	trans- caryophyllene	24.926	0.94-3.93	0.50-1.49
**17**	β-bisabolene	28.530	0.00-0.00	0.00-0.61
**18**	caryophyllene oxide	31.397	0.35-2.24	0.12-1.43

Phytochemical variability of essential oils of two Balkan en-demic species *Satureja pilosa* Velen. and *S. kitaibelii* Wierzb. ex Heuff. (Lamiaceae)

Table S2

Constituents and concentrations of *S. kitaibelii* (*S. montana* subsp. *kitaibelii)* populations in Bulgaria and Serbia.

**Number of constituents**	**Volatile constituents**	**RTime**	**Concentration Range. % (min-max) *S. montana ssp kitaibelii***	Concentration Range. % (min-max) ***S. montana***
**1**	α-pinene	6.045	0.00-5.67	0.56-0.91
**2**	camphene	6.454	0.00-2.95	0.14-0.24
**3**	oct-1-en-3-ol	7.262	0.00-1.17	1.60-1.87
**4**	myrcene	7.629	0.00-2.25	0.00-0.43
**5**	α-terpinene	8.514	0.00-3.16	0.91-0.94
**6**	p-cymene	8.813	1.11-29.22	16.60-26.06
**7**	unknown	8.944	0.00-26.24	0.00-0.00
**8**	cis-β-ocimene	9.205	0.00-8.53	0.00-0.00
**9**	trans-β-ocimene	9.532	0.00-9.31?	0.00-0.00
**10**	γ-terpinene	10.017	0.00-9.07	0.00-1.29
**11**	unknown	10.337	0.00-19.63	0.76-1.16
**12**	unknown	11.530	0.00-50.53	0.59-0.72
**13**	endo-borneol	14.302	0.73-12.50	0.49-1.31
**14**	terpinen-4-ol	14.755	0.00-16.69	0.46-1.31
**15**	trans-dihydrocarvone	15.760	0.00-3.58	0.00-0.00
**16**	unknown	16.822	0.00-3.38	0.00-0.00
**17**	neral	17.295	0.00-2.93	0.00-0.00
**18**	carvacrol methyl ether	17.538	0.00-2.86	0.00-0.00
**19**	thymoquinone	17.760	0.00-32.47	0.61-4.57
**20**	geraniol	18.011	0.00-54.55	0.00-0.00
**21**	geranial	18.562	0.00-6.00	0.00-0.00
**22**	bornyl acetate	19.257	0.00-5.77	0.00-0.00
**23**	thymol	19.958	0.00-29.65	17.33-23.02
**24**	carvacrol	20.303	0.00-10.11	39.00-46.21
**25**	geranyl acetate	23.374	0.00-33.04	0.00-0.00
**26**	β-bourbonene	23.480	0.00-4.21	0.00-0.00
**27**	trans- caryophyllene	24.986	1.30-6.09	1.31-1.74
**28**	α-humulene	26.288	0.00-4.23	0.00-0.00
**29**	γ-muurolene	27.401	0.00-23.07	0.00-0.00
**29**	unknown	28.016	0.00-3.07	0.00-0.00
**30**	β-bisabolene	28.570	0.00-5.41	1.50-1.81
**31**	spathulenol	31.234	0.00-6.52	0.44-1.80
**32**	caryophyllene oxide	31.445	0.00-8.88	0.97-1.48

Phytochemical variability of essential oils of two Balkan en-demic species *Satureja pilosa* Velen. and *S. kitaibelii* Wierzb. ex Heuff. (Lamiaceae)

Table S3

Locality, coordinates and used samples in population of *Satureja pilosa* and *S. kitaibelii* in Bulgaria and Serbia.

**Intrapopulation *Satureja pilosa***								**Distribution of *Satureja kitaibelii***				
**Stara planina (Balkan Mountains), Selce**				**Eastern Rhodope, Samokitka**								
**Samples**	**Coordinates**	**Masl**	**Samples /g**	**Samples**	**Coordinates**	**Masl**	**Samples /g**	**Samples**	**Coordinates**	**population**	**Masl**	**Samples /g**
**1**	42.63205°N 25.55463°E	686	12	**1**	41.40874°N 25.44305°E	608	130	**1**	42.959912°N 25.416690°E	K1	604	50
**2**	42.63198°N 25.55458° E	761	15	**2**	41.40874°N 25.44305°E	608	85	**2**	42.959810°N 25.416688°E	K2	587	50
**3**	42.63196°N 25.55461°E	761	12	**3**	41.40887°N 25.44288°E	609	40	**3**	42.959810°N 25.416688°E	K3	587	50
**4**	42.63195°N 25.55440° E	761	40	**4**	41.41005°N 25.44057°E	609	35	**4**	42.977192°N 23.135932°E	Bp	811	50
**5**	42.63204°N 25.55462° E	742	20	**5**	41.41004°N 25.44032°E	622	20	**5**	42.994656°N 24.178839°E	Gm	422	70
**6**	42.63204°N 25.55462° E	743	35	**6**	41.41004°N 25.44032°E	622	35	**6**	42.889486°N 23.201442°E	G 1	751	50
**7**	42.62698°N 25.55724°E	704	30	**7**	41.40981°N 25.44034°E	622	70	**7**	42.889069°N 23.200610°E	G 2	848	50
**8**	42.62935°N 25.55872°E	708	30	**8**	41.40976°N 25.44028°E	619	30	**8**	43.088225°N 23.101149°E	PP	1103	50
**9**	42.63189°N 25.55467°E	731	20	**9**	41.40981°N 25.44034°E	622	30	**9**	42.890742°N 23.159521°E	Bh	739	50
**10**	42.63184°N 25.5563°E	749	35	**10**	41.40978°N 25.44025°E	619	70	**10**	43.23394°N 22.29287°E	S1	326	75
**11**	42.63194°N 25.55452°E	749	20	**11**	41.40970°N 25.44010°E	616	40	**11**	43.25854°N 22.271807°E	S2	332	70
**12**	42.63189°N 25.55449°E	749	20	**12**	41.40977°N 25.43979°E	619	95	**12**	45.361046°N 19.659774°E	Sm	109	20

Beldie chan –Bh; Buchin prohod – Bp; Glojenski manastir - Gm; Gradec1 – G1; Gradec2 – G2; Kostenkovci 1 – K1; Kostenkovci 2 – K2; Kostenkovci 3 – K3; Prochoda Petrohan – PP; Serbia1 – S1; Serbia2 – S2; *S. montana* (cultivated) – Sm;
